# Transition from Self-Organized Criticality into Self-Organization during Sliding Si_3_N_4_ Balls against Nanocrystalline Diamond Films

**DOI:** 10.3390/e21111055

**Published:** 2019-10-28

**Authors:** Andrei Bogatov, Vitali Podgursky, Heinar Vagiström, Maxim Yashin, Asad A. Shaikh, Mart Viljus, Pradeep L. Menezes, Iosif S. Gershman

**Affiliations:** 1 Department of Mechanical and Industrial Engineering, Tallinn University of Technology, Ehitajate tee 5, 19086 Tallinn, Estonia; vitali.podgurski@ttu.ee (V.P.); heinar.vagistrom@taltech.ee (H.V.); mayash@ttu.ee (M.Y.); asadalamgir.shaikh@live.com (A.A.S.); mart.viljus@taltech.ee (M.V.); 2 Department of Mechanical Engineering, University of Nevada Reno, Reno, NV 89557, USA; pmenezes@unr.edu; 3 Joint Stock Company Railway Research Institute, Moscow State Technological University “Stankin” (MSTU “STANKIN”), 3rd Mytischinskaya Street 10, 129851, 127994, GSP-4, Moscow, Vadkovsky lane 1, Moscow, Russia; isgershman@gmail.com

**Keywords:** NCD, friction, self-organized criticality, self-organization

## Abstract

The paper investigates the variation of friction force (*F_x_*) during reciprocating sliding tests on nanocrystalline diamond (NCD) films. The analysis of the friction behavior during the run-in period is the focus of the study. The NCD films were grown using microwave plasma-enhanced chemical vapor deposition (MW-PECVD) on single-crystalline diamond SCD(110) substrates. Reciprocating sliding tests were conducted under 500 and 2000 g of normal load using Si_3_N_4_ balls as a counter body. The friction force permanently varies during the test, namely *F_x_* value can locally increase or decrease in each cycle of sliding. The distribution of friction force drops (*dF_x_*) was extracted from the experimental data using a specially developed program. The analysis revealed a power-law distribution *f^−µ^* of *dF_x_* for the early stage of the run-in with the exponent value (*µ*) in the range from 0.6 to 2.9. In addition, the frequency power spectrum of *F_x_* time series follows power-law distribution *f^−α^* with *α* value in the range of 1.0–2.0, with the highest values (1.6–2.0) for the initial stage of the run-in. No power-law distribution of *dF_x_* was found for the later stage of the run-in and the steady-state periods of sliding with the exception for periods where a relatively extended decrease of coefficient of friction (COF) was observed. The asperity interlocking leads to the stick-slip like sliding at the early stage of the run-in. This tribological behavior can be related to the self-organized criticality (SOC). The emergence of dissipative structures at the later stages of the run-in, namely the formation of ripples, carbonaceous tribolayer, etc., can be associated with the self-organization (SO).

## 1. Introduction

Amonton’s law states that friction force is linearly related to the normal load:

(1) $$F_{x}=kF_{z}$$

where *F_x_* is friction force, *k*—coefficient of friction (COF) and *F_z_*—normal load. On the other hand, friction is a complex phenomenon involving a number of processes, including deformation and fracture of surface asperities, adhesion, abrasion, chemical interactions, tribolayer formation, etc. These processes contribute to friction at different scales (nano-, micro- and macroscale) and hierarchy levels (molecular, surface asperity, component) [1]. Therefore, the frictional systems are essentially not strictly linear, because the COF may depend on many factors including normal load.

The energy (*W*) induced by external loading (mechanical work) is accumulated into the tribosystem (Δ*E*) and converted into the heat (*Q*).

(2) W=Q+ΔE,

The accumulated energy can be consumed by different processes including wear of material and generation of self-organizing (SO) dissipative structures (DS). SO with DS can influence the friction and wear [2].

Entropy production (∂S/∂t) is a useful concept for characterization of friction and wear, which are dissipative and irreversible processes. The entropy production of a system is the sum of the entropy production of processes occurring within the system. Moreover, according to the second law of thermodynamics, it is impossible to distinguish a part of the system at the macro-, micro- or nanoscale, in which the entropy production would be negative [3]:

(3) $$\frac{d_{i}S}{dt}=\sum_{n}\frac{d_{i}S_{nmicro}}{dt}=\sum_{n}\frac{d_{i}S_{nnano}}{dt}.$$

In systems far from thermodynamic equilibrium, entropy production can decrease due to SO with formation of DS. SO with DS can occur only after loss of thermodynamic stability [4]. The stability conditions for the thermodynamic system are given in the variational form by

(4) $$\frac{\partial }{\partial t}\left(\delta^{2}S\right)\geq 0,$$

where *δ*^2^*S* is the second variation of entropy. When Equation (4) is not fulfilled, SO can occur [5]. The SO is often characterized by the formation of a thin tribolayer at the friction surface [2]. It is estimated that more than 90% of the externally induced energy (Δ*E* in (2)) can be accumulated within the film surface [2,6]. In our previous work, the sliding of nanocrystalline diamond (NCD) films against Si_3_N_4_ balls was investigated [7]; it was shown that the initial polishing mechanism could not be related with SO, and SO can occur only after a run-in period. The formation of carbonaceous tribolayer, regular ripple patterns on the film surface and plastic deformation of the entire film during sliding, are examples of the SO structures [8].

Experimental and theoretical studies show that there are systems with many degrees of freedom, which can evolve into self-organized critical (SOC) state. This state is characterized by the release of energy through rapid relaxation processes (avalanches). The relation between the avalanche size (*A*) and the number of avalanches of the same size (*n*) can be described by the power law:

*n* = *A*^−*µ*^, (5)

where *µ* is an exponent characterizing the distribution of avalanches with different sizes [9,10]. A well-known example of the SOC system is a sand pile model [11] where grains of sand are randomly placed into a pile until the slope reaches a critical value. Further addition of grains triggers an avalanche affecting adjacent grains. Nosonovsky et al. [5] state that unlike SO systems, SOC systems are constantly “tuned” to a state whereby an avalanche can be initiated again.

Another example of the SOC system is a stick-slip friction between rubbing surfaces [9,12,13]. An indication of SOC is the power-law distribution of friction force drops (*dF_x_*) [9]. Some authors suggest strengthening conditions for SOC, namely the presence of frequency power spectra of *F_x_* time series and the existence of stationary state at long times [12,14].

The wear on diamond films is a load- and velocity-dependent process [15,16]. The most influential factors are anisotropy of mechanical and tribological properties [17], the ambient atmosphere [18,19], surface morphology [20,21], etc. It was suggested that due to considerable shear stress present at the diamond‒counter body interface, it becomes energetically more favorable for the diamond bonding to rehybridize to the sp^2^ configuration [22,23], thus contributing to formation of an amorphous carbonaceous lubricating tribolayer [15,24]. The surface morphology of diamond films plays a vital role during the run-in period in sliding wear tests, namely surface asperities interlock with counter body surface asperities, resulting in material fracture followed by micro-plowing and self-polishing wear mechanisms on the diamond film surfaces [20,21,25,26].

It is worth mentioning the hierarchical character of the wear of the diamond films. The Hertz contact pressure is calculated on the macro-level (i.e., mm), corresponding to the contact tribology [27]. However, different processes occurring on contact spots (hot-spots) between the ball and surface asperities are aspects of the asperity tribology (micro-level, i.e., µm) [27]. The characteristic sizes of ripple patterns observed in wear tracks on diamond films are in nanometer to micrometer ranges, as the length of a ripple can reach several microns and more, and the height is in nm range [8,26]. Formation of an amorphous carbonaceous lubricating layer due to stress-induced mechanochemical amorphization [15] and passivation of dangling bonds of carbon atoms produced during sliding by species from the ambient environment [14] occur on the atomic scale.

The present study aims to investigate the distribution of the friction force drops obtained after sliding tests with Si_3_N_4_ balls against nanocrystalline diamond (NCD) films grown on single-crystalline diamond SCD(110) substrates (NCD/SCD(110)). It allows us to carry out a more detailed analysis of friction behavior during the initial run-in period and to show early stages of formation of dissipative structures.

## 2. Materials and Methods

The NCD films (samples n2–n9) were grown on the SCD(110) substrates using the microwave plasma-enhanced chemical vapor deposition (MW-PECVD) method, see details in our previous study [28]. The coating thickness was 2.2 (samples n4 and n5), 8 (n6 and n7), 10.5 (n2 and n3), 14 (n9) and 22.5 µm (n8), respectively (Table 1). The root mean square surface roughness (Sq) was evaluated using atomic force microscopy (Bruker MultiMode^®^ 8 AFM). Scanning electron microscopy images were obtained using Zeiss EVO^®^ MA 15 system with LaB_6_ cathode in secondary electron mode, accelerating voltage 15 kV, working distance 5–7 mm.

Reciprocating sliding tests [29] were performed by means of CETR^®^ UMT-2 tribometer using Ø3 mm Si_3_N_4_ balls (surface roughness Ra = 0.012 μm, data provided by the manufacturer, REDHILL^®^). The displacement distance was 1 mm, frequency 5 Hz, and normal load was 500 and 2000 g (Table 1). The duration of tests was 600, 1200, 1800, 7200 and 14,400 s. Tests were conducted at room temperature and relative humidity of 35%.

**Figure 1:**
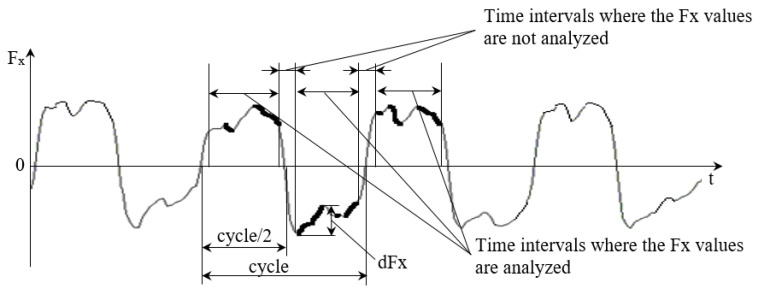
Example of friction force (*Fx*) behavior in reciprocating sliding test and data selection used in analysis.

Tangential or friction (*Fx*) and normal (*Fz*) forces, displacement distance, duration of the test, and COF values were recorded in a text file in the course of the reciprocating sliding test. An example of *Fx* behavior in a reciprocating sliding test is shown in Figure 1. Friction force drops are marked with a bold line on the plot. The number and size of *Fx* drops can differ per each half cycle. Because the direction of motion changes periodically, the friction force sign changes every half cycle. Therefore, friction force values measured in the vicinity of the points where the velocity is equal to zero should be excluded from the analysis (Figure 1). In the present study, the main focus is on the distribution of smaller *dFx* values. However, the width of the excluded time intervals can influence only the number of the highest *dFx* values. In other words, the analysis did not reveal the dependence of the distribution of *Fx* drops on excluded time interval width.

The frequency power spectrum of *Fx* time series was calculated using Statistica^®^ software.

## 3. Results and Discussion

**Figure 2:**
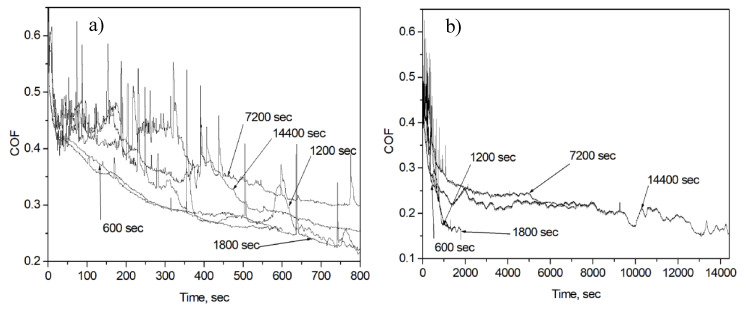
Coefficient of friction (COF) vs. time curves obtained on sample n3. Test duration is 600, 1200, 1800, 7200 and 14,400 s: first 800 s (**a**) and full-length (**b**).

Figure 2 shows COF vs. time curves obtained on the NCD/SCD(110) specimen n3. The shape of the curves differs for different tests, although no test conditions were changed. The reason for the variation of the shape of the COF curves was discussed in our previous work [7]. It was found that two typical types of COF vs. time curves are observed in sliding tests on the diamond films at room temperature due to bifurcation occurring after the initial run-in. The number of spikes of different intensity is particularly high during the first 800 s of sliding indicating frictional instabilities.

**Figure 3:**
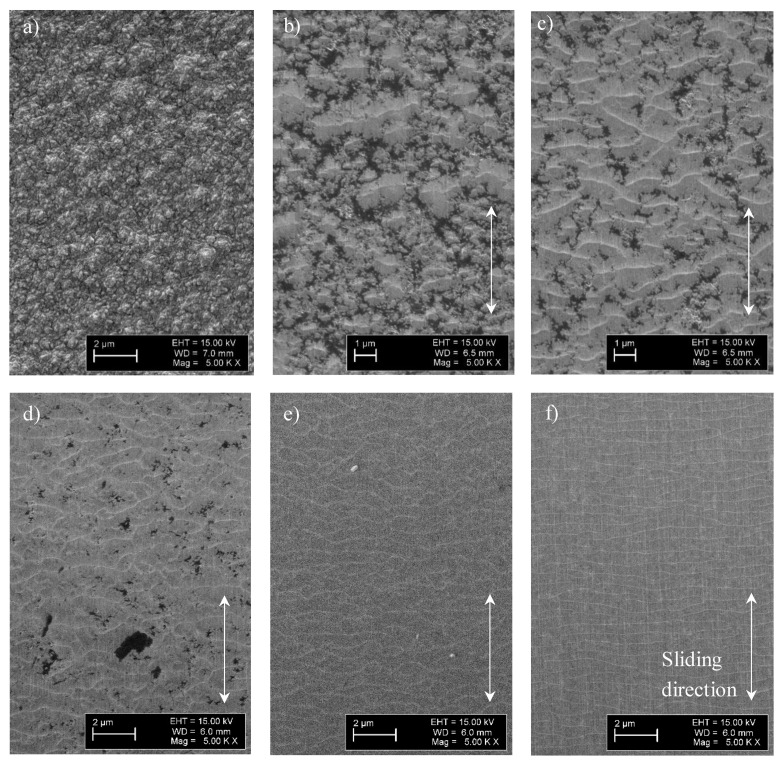
Ripple patterns observed on sample n3: pristine surface (**a**) and wear scars surface after the tests with the load of 2000 g and sliding time of 600 (**b**), 1200 (**c**), 1800 (**d**), 7200 (**e**) and 14,400 s (**f**).

Investigation of the surface of the wear scars revealed the formation of ripple patterns within the wear scars similar to ones observed in our previous studies [7,26]. A specific interest of the present investigation was an estimation of the initial sliding period when the first ripples start to form on the surface. Therefore, shorter sliding tests were carried out as well. Figure 3 shows SEM images taken of the surface of the wear scars after the sliding tests on sample n3. The duration of the tests was between 600 and 14,400 s, and the normal load was 2000 g. A network of ripples was observed after the first 600 s of sliding and for all following stages of the sliding regardless of the test duration. No fragmentation of the network into isolated ripples as well as disappearance of ripples was found. Formation of some ripples on top of the surface asperities can probably be expected before 600 s of sliding as well. Smoothening of NCD surface and formation of the continuous network of ripples on the surface was found after 1800 s of sliding (Figure 3d). There is an influence of the test duration on the shape and density of the ripples, compare for instance Figure 3c,e. It is worth stressing that the formation of ripple patterns is a continuous process, i.e., new patterns continuously formed in the course of the increase of the wear scar depth. There is a difference between the ripple pattern morphology observed in the present study and on the NCD films prepared on Si(100) substrate [7]. Namely, a network of interconnected ripples that was formed at the early stage of sliding was finally disintegrated into isolated ripples at the later stage. The understanding of the mechanisms causing morphological changes during sliding needs further research, which should include development of the self-organization theory, estimation of the influence of substrate properties and the structure of the diamond films.

**Figure 4:**
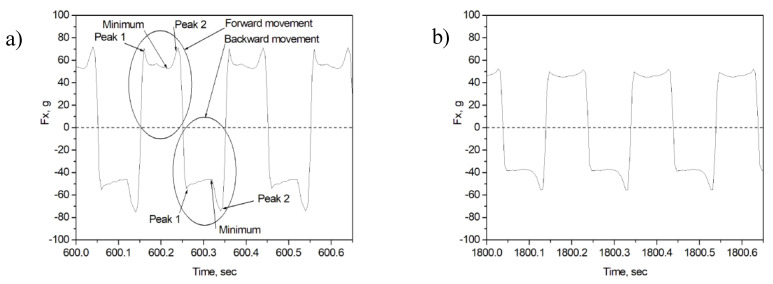
*Fx* vs. time curves recorded in the test with 2000 g of load after 600 (**a**) and 1800 s (**b**) of sliding on sample n3.

The characteristic behavior of *Fx* value during a sliding cycle is shown in Figure 4. A common feature is the existence of *Fx* peaks at the beginning (peak 1) and the end (peak 2) of the half cycle. The peak at the beginning is due to the fact that the coefficient of static friction is always greater than the coefficient of sliding friction. In most cases, the height of peak 1 is smaller than that of peak 2, and the height of both peaks is highest for the initial period of the run-in, i.e., for 0–600 s of sliding. The height of both peaks continuously decreases for longer periods of sliding and finally remains approximately constant after 1800 cycles. The variation of *Fx* value indicates stick-slip motion during sliding (Figure 1 and Figure 4).

**Figure 5:**
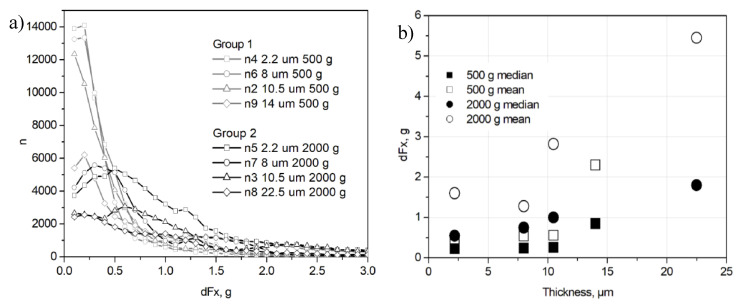
Distributions of *dFx* obtained for the first 600 s of the 1800 s length tests with the load of 500 and 2000 g (**a**) and median and mean values of the same distributions (**b**).

**Figure 6:**
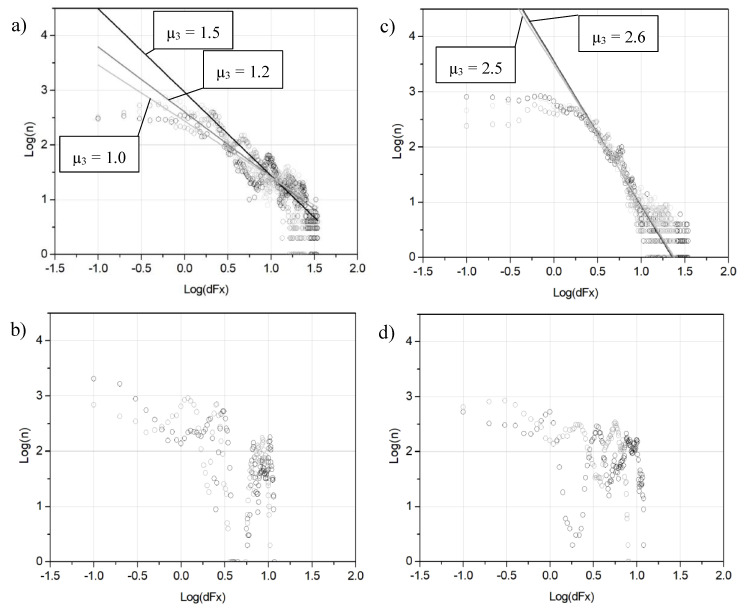
Log-log distributions of *dFx* for samples n3 and n5 (10.5 and 2.2 µm) after the next periods of sliding: n3 0–600 s (**a**), n3 6600–7200 s (**b**), n5 0–600 s (**c**) and n5 6600–7200 s (**d**). The data were obtained after 1800, 7200 and 14,400 s tests with 2000 g of load.

The distribution of *dFx* was extracted from the experimental data (Figure 5). The distributions obtained after the tests with 2000 g of load are shifted to the higher values of *dFx* in contrast to the distributions for 500 g of load (Figure 5b). This could indicate the existence of a threshold in the stick-slip system, which resembles the threshold for a sand pile slope angle in the “sand pile model” when a sudden avalanche occurs [5]. The type of distributions of *Fx* drops (or *dFx*) was estimated for different periods of sliding tests. For all samples under investigation (n2–n9), the behavior of log-log plots for the initial run-in period (0–600 s) was linear, i.e., it follows power-law distribution *f^−µ^* with exponent *µ*, see examples for samples n3 and n5 (Figure 6a,c). The *µ* value varied in the range of 0.6–2.9. This may indicate a presence of SOC in dry sliding friction between NCD and Si_3_N_4_ during the initial run-in period [9]. However, the log-log distributions of *dFx* obtained for other time intervals do not show power-law behavior, see examples for the period from 6600 to 7200 s of sliding (Figure 6b,d).

**Figure 7:**
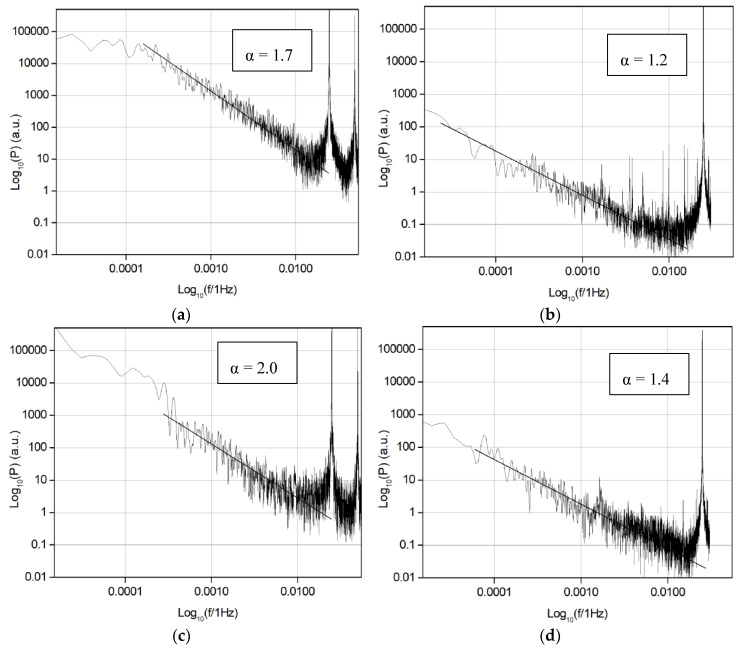
Frequency power spectra for samples n3 and n5 (10.5 and 2.2 µm). The data obtained after the 14,400 s tests with 2000 g of load and analyzed for the next periods of sliding: n3 0–600 s (**a**), n3 6600–7200 s (**b**), n5 0–600 s (**c**) and n5 6600–7200 s (**d**).

**Figure 8:**
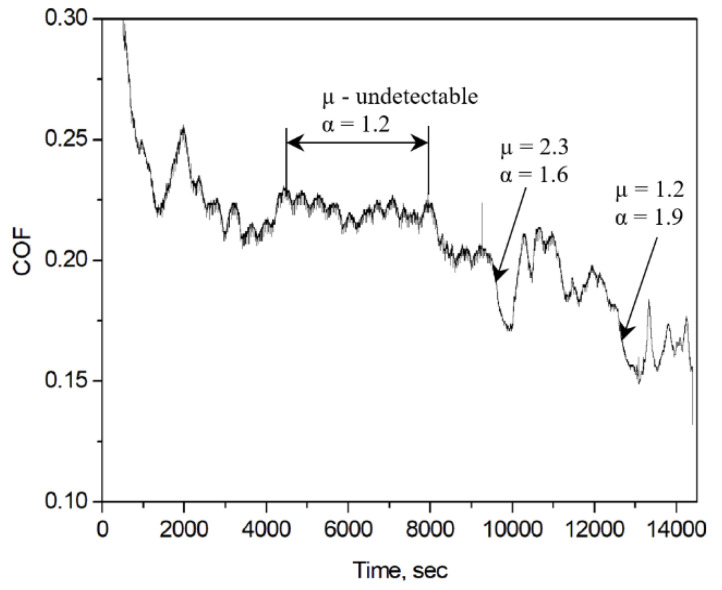
COF vs. time curve obtained on sample n3 (test for 14,400 s shown in Figure 2) and the values of exponents *µ* and *α* for different periods of sliding.

The frequency power spectrum density of *Fx* time series was calculated as well, and the results are shown in Figure 7. The *f^−α^* behavior was found with *α* value between 1.0 and 2.0. Values in the range of 1.5–2.0 were found for the initial run-in (0–600 s) and periods with a fast decrease of the COF value (Figure 8). These values are in good agreement with data in the literature [9]. For the sliding period between 600–1200 s, the α value varies in the range of 1.0–2.0, which is different from the period corresponding to the first 600 s of sliding. It was also found that the α value was about 1.0–1.2 for the sliding period, where the COF value only slightly varied, i.e., during a steady-state period of sliding (Figure 8). For some periods of sliding the *f* ^−*α*^ behavior was undetectable.

The power law distribution of *dFx* can be observed after the initial run-in period as well, namely for the time intervals where the COF value notably changes, probably due to instabilities during sliding. For instance, two characteristic periods with a relatively fast decrease of the COF value can be found between 9500–10,000 and 12,000–13,000 s of sliding (Figure 8). Therefore, the tribological system can exist in a SOC state.

**Figure 9:**
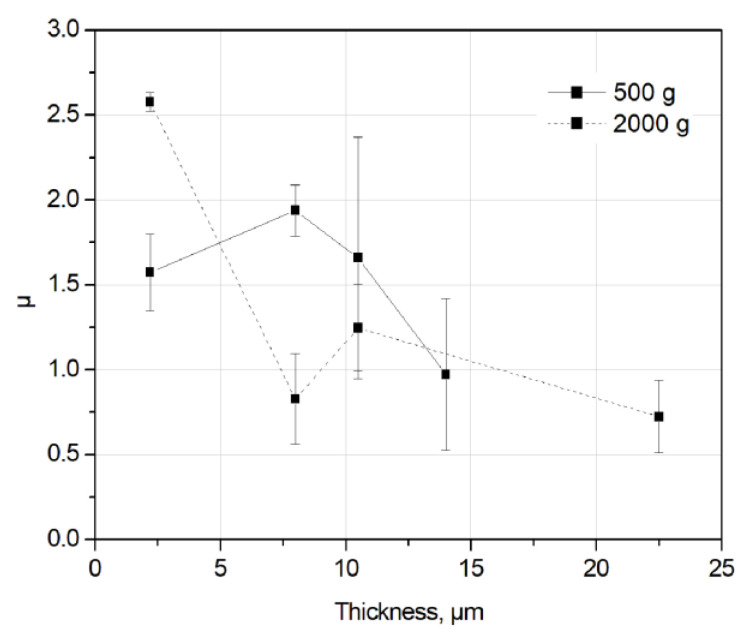
Averaged exponent *µ* vs. coating thickness for the run-in period (0–600 s).

**Figure 10:**
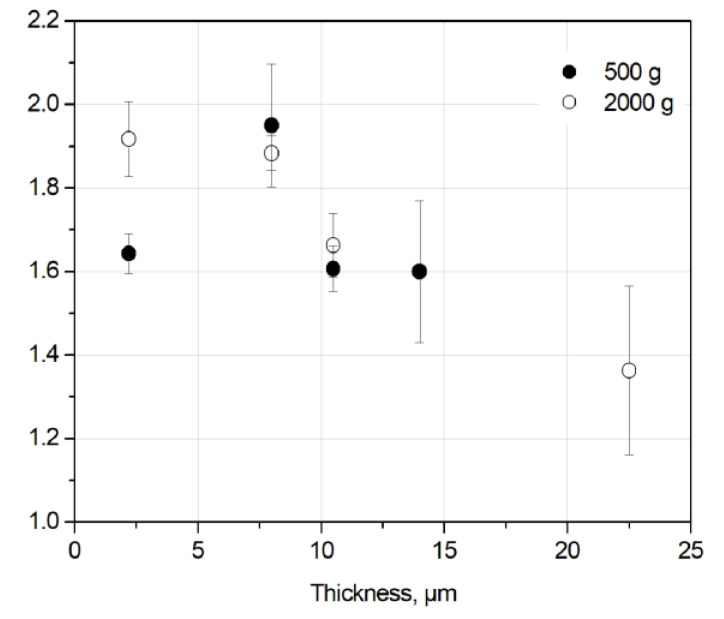
Averaged exponent α vs. coating thickness for the run-in period (0–600 s).

The averaged exponent *µ* estimated for the initial run-in period (0–600 s) is shown in Figure 9. The statistics are obtained on samples (n2–n9) after the sliding tests with different durations; however, only the first part of sliding curves (i.e., first 600 s) were taken into account. The *µ* value tends to decrease with increasing coating thickness. The roughness of the thicker films is higher than that of thinner ones (Table 1) and the value of friction drops increases with the increase of film thickness (Figure 5b). In other words, mechanical interlocking between counter body asperities is a crucial aspect for the understanding of SOC, in agreement with the conclusion by Zypman et al. [12]. The *α* value tends to decrease as well with increasing film thickness or increasing surface roughness (Figure 10).

However, the results of the present work differ in some aspects from the results obtained by Zypman et al. [12]. Namely, no power law *dFx* distribution was found during the steady-state sliding period. This discrepancy can be explained by formation of dissipative (self-organized) structures on the surface of NCD films during sliding. No tribolayer formation and effect of self-organization were discussed in the mentioned study, i.e., the tribological system investigated in the present work differs from one investigated by Zypman et al. [12].

It was mentioned in the Introduction that in order to initiate SO the system must loose thermodynamic stability. The dissipated energy needed to start up this process can be high. In the present study, SOC can be associated with a process that triggers SO. SOC can occur locally to dissipate more energy within a particular place on the surface. The initial run-in period can be characterized by fracture of asperities and self-polishing wear mechanisms, assuming that morphological changes first occur on top of the largest surface asperities, i.e., the dissipation of friction energy takes place locally. This can facilitate formation of DS. The evidence of local formation of ripples on the top of diamond grains can be found in our previous study on microcrystalline diamond films [30]. In other words, the ripples as an indication of DS form at the places where initial SOC effect took place locally. In the course of sliding, SOC occurs subsequently at the smaller asperities, which finally leads to the formation of ripple patterns on the entire surface of the wear scar. Carbonaceous tribolayer forms during these processes, which leads to a lower COF value and smoother sliding for the steady-state period of the tests (compare Figure 2a,b).

The SOC and SO can be distinguished by a different way of releasing mechanical energy induced by external work. SOC is related to the process of energy release through the avalanches, however, dissipation of energy through the formation of tribolayer, ripples formation, etc., on the surface occurs in the case of SO. SOC is characterized by the passage of processes with a positive production of entropy, i.e., the production of entropy increases. SO is characterized by the passage of processes with negative entropy production, i.e., entropy production decreases. In addition, there is a subtle difference between the SOC and SO. As was mentioned in the Introduction, the SOC system is permanently “tuned” to a state whereby an avalanche can be initiated again. In the case of SO with DS, it was found that the formation of ripple patterns is a permanent process, i.e., they are formed at different depths of the wear scars; however, no time gap exists during the sliding when there are no ripples on the wear scar surface. The formation of a new carbonatious tribolayer is a continuous repeatable process as well [31]. In the present work, the SOC corresponds to initial fracture and polishing of materials or severe wear. In the case of SO, the formation of DS accumulates energy; therefore, only a part of external work is consumed by wear.

Furthermore, it is worth comparing mechanical interlocking with the seizure, which was considered as unconstructive SO in our previous study [7]. A seizure is related to the possibility of the system stopping sliding and was related to an increased adhesion between the counter bodies [2]. SOC investigated in the present study can be related to the mechanical interlocking between counter body asperities, and there is a threshold in stick-slip when the body cannot move. Therefore, it demonstrates the inherent similarity between mechanical interlocking (SOC) and seizure (SO). In addition, it was found that adhesive wear is probably the leading wear mechanism after the run-in period [7], i.e., the wear mechanisms like fracture and initial polishing occurring at the initial run-in (see Introduction) continuously change into adhesive wear.

## 4. Conclusions

The distribution of friction force drops during reciprocating sliding on NCD/SCD(110) samples against Si_3_N_4_ balls was analyzed. The power-law type of friction force drops distribution was observed at the initial run-in period of the sliding tests, i.e., during the first 600 s of sliding. This indicates the presence of SOC. The value of exponent *µ* depends on the roughness of the NCD films. The formation of dissipative structures starts during later stages of the run-in period, i.e., SOC continuously changes into SO. The SOC was not found during the steady-state regime of sliding with an exception for periods where instabilities in sliding occur.

The transition from SOC during the run-in period of sliding to SO during the steady-state period observed in the present study indicates an existence of a class of tribological systems with specific behavior. It differs, for instance, from systems where SOC was found for the steady-state period of sliding.

## Figures and Tables

**Table 1:** Thickness and surface roughness of samples and normal loads used in the tribological tests.

Sample	n4	n5	n6	n7	n2	n3	n9	n8
Normal load [g]	500	2000	500	2000	500	2000	500	2000
Coating thickness [µm]	2.2		8		10.5		14	22.5
Roughness Sq [µm]	33		55		56		66	78
